# Evaluation of Gait Variable Change over Time as Transtibial Amputees Adapt to a New Prosthesis Foot

**DOI:** 10.1155/2019/9252368

**Published:** 2019-05-20

**Authors:** Xueyi Zhang, Goeran Fiedler, Zhicheng Liu

**Affiliations:** ^1^School of Biomedical Engineering, Capital Medical University, Beijing, China; ^2^Department of Rehabilitation Science and Technology, University of Pittsburgh, Pittsburgh, PA, USA; ^3^Beijing Key Laboratory of Fundamental Research on Biomechanics in Clinical Application, Capital Medical University, Beijing, China

## Abstract

A variety of prescribed accommodation periods have been used in published prosthesis intervention studies that have examined biomechanical outcomes. Few investigators included repeated measurements in their study design, leaving questions as to how measured outcomes change as amputees acclimate to a new prosthesis. This paper is the product of our investigation as to whether measured gait variables were affected by the duration of accommodation period, and to assess the relationship between measured outcomes and the subjective perception of the participants. A sample of transtibial amputees were recruited for this study. Gait data was collected by wearable sensor repeatedly, starting immediately after fitting the interventional foot and extending over a subsequent four days. Participants indicated their perceived accommodation quality on a visual analog scale (VAS). A total of twelve commonly used spatiotemporal gait parameters were analyzed. Friedman tests were used to determine overall differences across time points in both early (one hour) and late (day two through five) accommodation phases, for each gait variable. Statistically significant changes across the early phase were found for variables gait speed *χ*^2^(2)=8.000,* p*=0.018, cadence *χ*^2^(2)=7.185,* p*=0.028, and double support time on the sound side *χ*^2^(2)=8.615,* p*=0.013. Across days two through five, no gait variable significantly changed. VAS scores correlated strongly with step count (r=1.000,* p*<0.001) and cadence (r=0.857,* p*=0.014). Longer accommodation periods resulted in less deviations of gait variables for the clinical assessment in the process of prosthetic rehabilitation. Trying out prosthetic interventions for less than one hour has yielded unreliable outcomes.

## 1. Introduction

Biomechanical examinations of prosthesis users are important in providing clinicians useful information on measurement outcomes to improve their patient's function, and research is frequently conducted to assess the effectiveness of new prosthetic components with regards to users' safety/stability, energy expenditure, activity [1–4], and other factors. In this context, providing subjects with adequate accommodation time is important to ensure that they have properly acclimated to any intervention prior to assessment. Otherwise, outcomes may be affected by their unfamiliarity with the devices and may not reflect real-life situations after long-term use. However, evidence on the required accommodation period after a fitting of a new prosthesis is limited in the published literature.

Accommodation times provided prior to assessing a tested prosthetic component's effects vary widely among studies [5], ranging from zero (i.e., testing immediately after receiving a new prosthesis) to several months. While most authors just report an accommodation period without offering an explanation for the selected time [6–8], some do provide rationales for their selection. Explanations that have been offered include the following:

- The accommodation period chosen was based on clinical experience and judgment of most amputees [9]
- As the accommodation time of a particular component has not been reported in published literature, and a fixed time for acclimation to a particular component was not set [10]
- Instead of clinical judgment, investigators set up their own criteria, such as proficiency in performing defined use tasks during recruitment and enrollment section [11]
- Provided accommodation time was justified by citing a case study [12] published in 1995 [1, 13, 14].

Regardless of the approach, justifying the same adaptation time for all subjects across a study sample is challenging since individual abilities to accommodate are likely different.

Notwithstanding these challenges, the current lack of evidence on how much accommodation time is appropriate is one of the threats to the validity of findings and efficiency of protocol design in prosthetics research. To the authors' knowledge, there is limited published work that describes systematic measurements of gait parameters to investigate accommodation time. English et al. deemed that at least one week is required to let pertinent gait parameters stabilize, while three weeks are preferable. However, the general applicability of these conclusions is limited, as they were based on data from just one patient, and taking only part of the available variables into account. Another paper on the effects of adaptation to a new prosthetic knee joint reported gait variables on two measured time points, immediately upon fitting and after conclusion of the learning process [15]. Findings suggest that “a few hours of adaptation time are sufficient if the motion patterns required are similar to that of the previous fitting” and that longer periods should be allowed if “specific functions require the learning of a motion” [15]. However, the assessments were limited to two separate dates only, neglecting possible changes of the movement patterns across the whole adaptation period.

The goal of our work was to investigate whether individually varying accommodation times are reflected in measurable gait variables. We hypothesized that changes in gait become smaller as subjects get better accustomed to prosthetic interventions. We further investigated how gait variables (cadence, step length, double support time, etc.) are correlated to subjects' perceived rate of accommodation.

## 2. Methods

### 2.1. Subjects

A sample of transtibial amputees with energy storage and return (ESAR) foot were recruited for this Institutional Review Board approved study. Inclusion criteria were an activity classification of K2 to K4, with experience in prostheses use for more than one year. Individuals with existing skin damage or acute ailments that would restrict their usual mobility or prosthesis use were excluded from participation. Informed written consent was obtained prior to data collection.

### 2.2. Protocol Design

Data collection was spread out over five days in an effort to capture a most complete adaptation phase with a high number of assessments and short intervals between them. A systematic review, which aimed to identify evidence-based consensus of when biomechanical analysis on transtibial amputees should occur, reported the median number of days given for accommodation to a new prosthesis was five [16].

A multiaxial foot (1A30 Greissinger Plus, Otto Bock, Duderstadt, GER) was selected as the interventional foot because it was safe, markedly different from participants' original prosthesis foot (to induce a substantial adaptation process), and inexpensive. The “1A30 Greissinger Plus” provides stability on uneven ground; however, the original feet generally provided more energy return. The prosthetic alignment with the interventional foot was first optimized and documented by a credentialed prosthetist in a separate session at least seven days prior to the first day of data collection. In this session, the doffed prosthesis had been placed in an alignment device, where the position of socket and foot with respect to each other was documented by plumb lines on the socket, and shape and direction of the foot were documented on the alignment device. This preparation allowed data collection to begin immediately after eventually installing the interventional foot. This eliminated that fitting and alignment changes introduced confounding variables. The same study prosthetist performed all work on subjects' prostheses. Details of the protocol design have been described previously [17].

Gait data collected with the original prosthetic foot was set as the baseline (Table 3). Upon receiving the modified prosthesis, subjects were asked to walk along a 30-meter hallway and return the same way in their preferred gait speed, taking rest breaks as needed. Gait data was collected by a G-Walk sensor (BTS Bioengineering, Milan, ITALY) that has been utilized in various research studies [18–20]. This wireless device was positioned on the 5th lumbar vertebrae, secured by an ergonomic belt, allowing subjects' free body movement. Data was streamed to a computer via Bluetooth at a sampling frequency of 100 Hz. Short walk samples (approximately twenty steps) were collected repeatedly, starting immediately after fitting the interventional foot (T0), as well as after half an hour (T1) and one hour (T2) in Phase 1. On the subsequent four days, subjects were instructed to go about their routine activities as usual. Short gait data measurements (approximately twenty steps as in Phase 1) were scheduled in roughly 24 h increments over the next four days (D2-D5) (Phase 2). Concurrently, with each gait data collection, participants indicated their perceived accommodation quality on a VAS ranging from no accommodation at all on the left end to complete accommodation on the right end. The use of a VAS without numbers or tick marks had the aim to record the perceived accommodation quality independently between walking trials. A prosthesis-worn activity tracker (Up move, Jawbone, San Francisco, CA) was used to record step counts throughout the whole five-day intervention period. After the final data collection, the original state of participant's prosthesis, including their initial foot, was restored.

### 2.3. Gait Variables

A total of twelve commonly used spatiotemporal parameters, including gait speed (in m/s), cadence (steps/min), step length left/right (% gait cycle), stance time left/right (% gait cycle), swing time left/right (% gait cycle), double support time left/right (% gait cycle), and single support time left/right (% gait cycle), were extracted to examine whether measurement outcomes were affected by the duration of the accommodation period when prosthesis users adapted to a new prosthetic foot. These gait variables were analyzed by sound side (S) and affected side (A) for each time point.

### 2.4. Data Analysis

All statistical tests were carried out with IBM SPSS version 24. The significance criterion was set at 0.05. Given the small sample size, Friedman tests were used to determine overall differences across time points in both Phase 1 and Phase 2, for each gait variable. Post hoc analysis with Wilcoxon signed-rank tests was conducted with a Bonferroni correction applied that *α* was adjusted to 0.025 in Phase 1 and 0.017 in Phase 2. Coefficient of Spearman correlations between scores of VAS and measured outcomes of gait variables were computed. The strength of association was defined as weak (<0.50), moderate (0.50–0.80), or strong (>0.80). Phases 1 and 2 were then compared with each other based on the observed change in gait during each.

## 3. Results

Eight participants were recruited, of which seven completed the protocol and were included in this analysis (Table 1). There was overall a statistically significant difference across Phase 1 data points of variables gait speed (*p*=0.018), cadence (*p*=0.028), and double support time on the sound side (*p*=0.013) (Table 2). A Bonferroni correction for post hoc analysis resulted in a significance level set at 0.025. There was a reduction in gait speed between T1 and T2 (*p*=0.018), as well as an increase in double support time on the sound side (*p*=0.018). Across Phase 2, there were no significant differences between repeat measurements of gait variables (Table 3).

**Figure 1:**
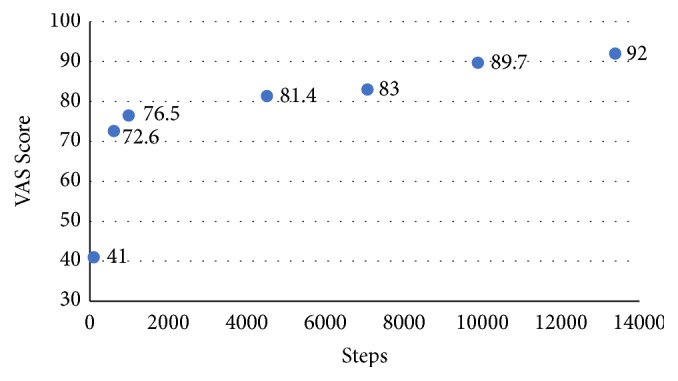
VAS scores and step counts while participants accommodated to a new prosthesis foot across the whole adaptation phase.

Table 4 shows the relationship between subjective perception of accommodation quality and objective measurement outcomes. Mean scores of perceived accommodation quality and steps measured on consecutive time points are presented in Figure 1. Perceived accommodation quality correlated strongly with step counts (r=1.000,* p*<0.001) as well as cadence (r=0.857,* p*=0.014).

## 4. Discussion

Biomechanical examinations of prosthetic interventions are important in providing clinicians useful information on outcomes to help improve their patients' function. The aim of this study was to investigate whether measurement outcomes were affected by the duration of the accommodation period when prosthesis users adapted to a new prosthetic foot, and to assess the relationship between measured outcomes and the subjective perceptions of the participants.

A recently published systematic review [21] of accommodation times noted that approximately 77.8% of transtibial prosthetics studies which tested subjects on the day of receiving a new prosthesis, tested them in the first hour. Our results (Table 2) indicate that trying out prosthetic interventions for less than one hour will yield unreliable outcomes. Indeed, statements regarding the limitations of short accommodation periods were often made in discussion sections of published papers [22–24]. Assessing intervention effects too early may limit the scientific and clinical significance of findings and make it difficult to compare results of different studies.

Even when the literature discussed any overall difference across the accommodation process, it remained unknown when and how exactly the gait pattern changed significantly. Our post hoc analysis showed a significant increase in double support time on the sound side between T1 and T2 (Table 2). Howcroft et al. [25] suggested that percent double support time was frequently related to clinical balance and mobility measures. If subjects have not properly acclimated to an intervention, they may be unsteady on their feet, trying to maximize the amount of time where both feet are on the ground. The prolonged stance time would especially apply to the sound side, providing the most stability while acclimating to the intervention. We also found a reduction in gait speed between T1 and T2 (Table 2). Gait speed is a valuable variable in energy expenditure tests and the found difference may indicate important differences in concomitant variables. A reduced gait speed would entail that participants might be uncomfortable with the intervention, which may be anticipated after a short adaptation time [22]. It is unclear to what extent those differences might change after longer adaptation.

**Figure 2:**
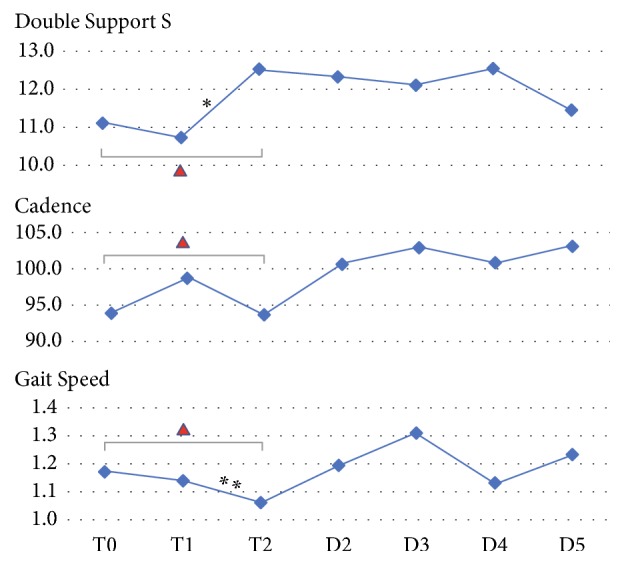
Trajectory across the entire adaptation phase of gait variables that significantly changed across Phase 1 (data is reported as mean). ▲ Repeated measures Friedman tests showed statistically significant differences in Phase 1 of gait variables.

In Phase 2, unlike in Phase 1, there were no significant differences in repeat measurements of gait variables (Figure 2). This confirms our hypotheses that longer accommodation periods result in less deviations. While it is arguably desirable to provide longer rather than shorter accommodation times in research studies, shorter periods may be necessary to reduce the burden of participation for subjects or to accommodate budget constraints. To that end, some investigators have introduced standardized training during the accommodation period [10, 23], trying to minimize deviations resulting from habit and enable participants to maximize the use of each new prosthetic component.

One aim of our analysis was to assess the strength of the relationship between objective measures of gait variables and subjective perceptions of accommodation. We found that step counts and cadence are most indicative of the quality of accommodation as perceived by prosthesis users. This is corroborated by our finding of significant changes in cadence during Phase 1. Overall, the score of perceived accommodation quality increased at about the same rate as step count from throughout the intervention period (Figure 2). This may indicate that the perceived accommodation quality increased along time and exercise. A similar finding was reported on the effect of prosthetic alignment, which was reflected both in measureable gait variables and in users' perceptions [26].

## 5. Limitations 

One limitation of this study is the small sample size. As is the case for many studies in P&O, the statistical power of ours was limited by a small sample size. A larger scale study has been motivated by the here presented preliminary findings and is currently underway.

Measured variables were inevitably limited in number. Likewise, the sample was very homogeneous, containing only male transtibial prosthesis users with ESAR feet. Thus, the results cannot be generalized to other important outcomes (i.e., energy expenditure) and populations. Further studies should include more variables and compare differences in amputation level and interventions across longer adaptation phases.

## 6. Conclusion

Trying out prosthetic interventions for less than one hour will yield unreliable outcomes. Longer accommodation periods result in less deviations of gait variables for the assessment in the process of prosthetic rehabilitation. Cadence, gait speed, and double support duration were found to be the most sensitive measures to capture changes in gait while transtibial prosthesis users accommodated to a new prosthetic foot.

## Figures and Tables

**Table 1:** Anthropometric data of the study sample.

Subject	Age /years	Height /cm	Weight /kg	Time since limb loss/years	Original foot
1	55	177	85.7	19	College Park Trustep
2	73	168	82.1	11	Otto Bock LuXon Max
3	62	183	88.5	7	Endolite Echelon
4	53	173	93.0	4	Ossur Talux
5	61	178	88.5	5	College Park Soleus
6	40	186	68.0	16	Otto Bock Triton Low Profile
7	27	170	90.7	22	Ossur Re-Flex

**Table 2:** Gait data on repeat measured time points in Phase 1 while subjects adapt to the new prosthetic foot.

Gait variables	T0	T1	T2	Friedman tests *χ*^*2*^(2)*, p* value	T0-T1	T1-T2
Gait Speed (m/s)	1.18 (1.00-1.28)	1.16 (1.00-1.26)	1.11 (0.85-1.21)	8.000, 0.018*∗*	0.499	0.018*∗*
Cadence (step/min)	95.6(87.3-107.4)	98.8 (86.9-109.2)	98.8(75.2-108.4)	7.185, 0.028*∗*	0.063	0.028
Step Length A (%)	53.4 (50.3-54.0)	51.0 (48.4-54.6)	50.5 (48.5-54.3)	0.286, 0.867		
Step Length S (%)	46.6 (46.0-49.7)	49.0 (48.4-51.6)	49.5 (45.7-51.5)	0.286, 0.867		
Stance Phase A (%)	58.4 (56.0-61.4)	59.7 (54.8-60.2)	61.9 (56.3-63.8)	3.714, 0.156		
Stance Phase S (%)	61.4 (57.6-67.6)	60.2 (58.5-66.6)	61.0 (56.3-63.8)	0.000, 1.000		
Swing Phase A (%)	41.6 (38.6-44.0)	40.3 (39.8-45.2)	38.1 (36.2-43.7)	3.714, 0.156		
Swing Phase S (%)	38.6 (32.4-42.4)	39.8 (33.4-41.5)	39.0 (31.0-42.2)	0.000, 1.000		
Double Support A (%)	10.6 (8.2-11.2)	9.2 (7.1-13.4)	12.1 (8.6-14.5)	3.429, 0.180		
Double Support S (%)	8.6 (7.3-17.0)	10.0 (7.3-11.7)	11.7 (7.8-16.2)	8.615, 0.013*∗*	0.892	0.018*∗*
Single Support A (%)	38.1 (33.6-41.6)	39.9 (33.4-42.3)	38.0 (30.8-42.1)	0.286, 0.867		
Single Support S (%)	41.5 (38.8-44.5)	40.6(39.8-45.3)	38.1 (36.3-44.1)	3.714, 0.156		

Median value (25^th^-75^th^ percentiles value), *∗p*<0.05.

T0: immediately after fitting the interventional foot, T1: half an hour, T2: one hour.

A: affected side, S: sound side.

**Table 3:** Gait data on repeat measured time points in Phase 2 while subjects adapt to the new prosthetic foot.

Gait variables	D2	D3	D4	D5	Friedman tests *χ*^*2*^(3)*, p* value	Baseline
Gait Speed (m/s)	1.13 (1.06-1.34)	1.29 (1.21-1.30)	1.12 (0.95-1.30)	1.19 (1.08-1.49)	4.433, 0.218	1.17(0.83-1.35)
Cadence (step/min)	99.8(93.0-110.7)	103.0 (94.2-109.7)	101.9(89.5-110.8)	104.3(96.1-107.1)	5.118, 0.163	100.9(85.6-105.5)
Step Length A (%)	51.3 (48.7-52.6)	51.7 (49.7-55.5)	51.2 (49.8-54.6)	49.9 (47.6-54.1)	2.294, 0.514	51.5(49.3-53.8)
Step Length S (%)	48.7 (47.4-51.3)	48.3 (44.5-50.3)	48.8 (45.4-50.2)	50.1 (45.9-52.4)	2.294, 0.514	48.5(46.2-50.7)
Stance Phase A (%)	60.5 (55.8-63.5)	60.5 (57.9-62.8)	59.4 (56.1-63.4)	59.2 (57.9-63.2)	1.478, 0.687	62.1(56.0-65.8)
Stance Phase S (%)	60.0 (59.2-66.1)	62.9 (58.3-66.2)	65.7 (57.4-67.2)	62.6 (57.8-65.7)	0.882, 0.830	61.4(60.5-72.9)
Swing Phase A (%)	39.5 (36.5-44.2)	39.5 (37.2-42.1)	40.6 (36.6-43.9)	40.8 (36.8-42.1)	1.478, 0.687	37.9(34.2-44.0)
Swing Phase S (%)	40.0 (33.9-40.8)	37.1 (33.8-41.7)	34.3 (32.8-42.6)	37.4 (34.3-42.2)	0.882, 0.830	38.6(27.1-39.5)
Double Support A (%)	11.6 (7.8-13.1)	8.7 (7.2-15.8)	11.0 (8.5-14.5)	11.4 (7.8-14.7)	1.412, 0.703	10.6(8.1-16.3)
Double Support S (%)	11.6 (9.3-15.6)	11.5 (9.5-12.3)	11.7 (9.8-13.9)	10.6 (9.8-10.9)	6.529, 0.089	12.4(9.7-20.6)
Single Support A (%)	39.7 (35.7-40.8)	37.1 (34.7-41.6)	34.6 (32.6-43.0)	37.9 (34.6-42.5)	1.388, 0.708	38.1(26.7-40.4)
Single Support S (%)	39.2 (36.6-42.6)	39.2 (37.5-42.5)	40.1 (36.5-43.9)	41.3 (36.7-42.0)	0.529, 0.912	37.9(34.5-44.5)

Median value (25^th^-75^th^ percentiles value), *∗p*<0.05.

A: affected side, S: sound side.

**Table 4:** Relationships between perceived accommodation quality and gait variables of trans-tibial prosthesis users.

Gait Variables	Spearman Coefficient r (*p*-value)
Steps	1.000 (<0.001)*∗∗*
Gait Speed (m/s)	0.571 (0.180)
Cadence (step/min)	0.857 (0.014)*∗*
Step Length A (%)	-0.214 (0.645)
Step Length S (%)	0.214 (0.645)
Stance A (%)	0.250 (0.589)
Stance S (%)	0.500 (0.253)
Swing A (%)	-0.250 (0.589)
Swing S (%)	-0.500 (0.253)
Double Support A (%)	0.714 (0.071)
Double Support S (%)	0.464 (0.294)
Single Support A (%)	-0.286 (0.535)
Single Support S (%)	-0.321 (0.482)

A: affected side, S: sound side, *∗p*< 0.05. *∗∗p*< 0.001

## Data Availability

The data used to support the findings of this study are available from the corresponding author upon request.
